# Stereotactic Ablative Radiotherapy (SABR) in inoperable oligometastatic disease from colorectal cancer: a safe and effective approach

**DOI:** 10.1186/1471-2407-14-619

**Published:** 2014-08-27

**Authors:** Tiziana Comito, Luca Cozzi, Elena Clerici, Maria Concetta Campisi, Rocco Luca Emanuele Liardo, Pierina Navarria, AnnaMaria Ascolese, Angelo Tozzi, Cristina Iftode, Fiorenza De Rose, Elisa Villa, Nicola Personeni, Lorenza Rimassa, Armando Santoro, Antonella Fogliata, Pietro Mancosu, Stefano Tomatis, Marta Scorsetti

**Affiliations:** Radiotherapy and Radiosurgery Department, Humanitas Clinical and Research Center, Rozzano, MI Italy; Medical Physics Unit, Oncology Institute of Southern Switzerland, Bellinzona, Switzerland; Medical oncology and hematology unit, Humanitas Clinical and Research Center, Rozzano, MI Italy

**Keywords:** Liver, Lung, Colorectal oligometastases, RapidArc, Stereotactic ablative radiotherapy

## Abstract

**Background:**

To assess the safety and efficacy of Stereotactic Ablative Radiotherapy (SABR) in oligometastatic patients from colorectal cancer.

**Methods:**

82 patients with 1–3 inoperable metastases confined to one organ (liver or lung), were treated with SABR for a total of 112 lesions in an observational study. Prescription dose ranged between 48 and 75Gy in 3 or 4 consecutive fractions. Primary end-points were local control (LC), overall survival (OS) and progression-free survival (PFS). Secondary end-point was toxicity.

**Results:**

Median follow-up was 24 months (range 3–47). One, two and three years LC rate was 90%,80% and 75% (85%,75% and 70% for lung and 95%, 90% and 85% for liver metastases; no statistically significance was found). The difference in LC between the subgroup of lesions treated with ≥60 Gy (n = 58) and those irradiated with <60 Gy (n = 52) was statistically significant, with a 1, 2 and 3 yrs LC of 97%,92% and 83% for the higher dose, compared to 85%,70% and 70% for the lower dose (*p* < *0.04*). Median OS was 32 months. Actuarial OS rate at 1, 2 and 3 yrs was 85%,65% and 43%. Univariate analysis showed a correlation only between OS and cumulative GTV > 3 cm (*p* < *0.02*). Median PFS was 14 months, with a PFS rate of 56% at 1 yr and 40% at 2-3 yrs, without correlation with the site and prescription dose (*p* < *0.48* and *p* < *0.56*). No patients experienced radiation-induced liver disease or grade >3 toxicity.

**Conclusions:**

SABR is a safe and feasible alternative treatment of oligometastatic colorectal liver and lung metastases in patients not amenable to surgery or other ablative treatments.

## Background

The concept of “oligometastatic disease” was introduced to identify a condition in which the number and sites of metastases are limited from one to five [1]. According to this hypothesis of orderly progression, this is an intermediate state before widespread dissemination. Therefore, the local control of oligometastases could still improve the systemic control of the disease.

Conversely, studies suggested that oligometastases can represent only the clinically detectable lesions in the context of widespread occult disease and their treatment may not affect survival [2]. Presumably, both hypothesis are correct [3]. Given the improvements in diagnostic imaging, the prevalence of oligometastatic state is increasing [4].

Colorectal cancer (CRC) is one of the tumors that most often presents solitary recurrence or oligometastasis, commonly in the liver and lung [5]. The surgical resection is associated with a survival increase [6–11]. The hepatic resection can provide a 5-year overall survival (OS) rates of 37–58% [6, 7], as well as the pulmonary resection can provide a 5-year survival rate of 38–50% [9–11]. Approximately 70-90% of metastatic patients, however, are unresectable because of technical difficulties, unfavorable tumor factors or patients co-morbidities [7, 8, 10].

Other local approaches, such as radiofrequency ablation (RFA), have been used as alternative to surgical resection of CRC metastasis. Also these techniques presents some limitations related to the size and location of the target lesions [12–14].

The use of Stereotactive Ablative Body Radiation Therapy (SABR) was investigated in the treatment of oligometastasis with promising results, utilizing either a single dose or a small number of fractions [15]. The SABR approach has proved an effective treatment for inoperable liver and lung metastases [16–19], particularly in terms of local control (LC).

This prospective study examined patients with liver and lung oligometastases by colorectal cancer not amenable to surgery or other local treatments, treated with SABR by means of volumetric modulated arc therapy (in RapidArc, RA, form).

We hypothesized that for the setting of CRC patients, who are in this intermediate, potentially-curative oligometastatic state, the ablative radiation treatment of inoperable recurrences can represent an efficacy therapeutic option.

## Methods

### Patients selection

82 patients with 1–3 detectable metastasis from CRC, confined to one organ (liver or lung) were prospectively enrolled and treated with SABR between February 2010 and January 2013 according to the methods described in [18] in an observational, non-interventional study (performed with the approval of the Humanitas Cancer Center Ethical Review Committee and in compliance with the Helsinki Declaration) to assess the safety and effectiveness of SABR. SABR was prescribed by the radiation oncologist as part of standard care for these patients if presented: histologically proven colorectal adenocarcinoma, radical resection of the primary tumor, 1–3 lesions confined to one organ such as the liver or lung, assessed as inoperable (due to technical reasons or to concomitant co-morbidities as cardiac diseases) and not amenable to another local treatment, with a maximum tumor diameter less than 6 cm, no evidence of progressive or untreated gross disease outside the liver and lung, no prior radiation therapy to the targeted area, no concurrent chemotherapy, either within 14 days before SABR or until the first revaluation, normal liver volume greater than 1000 cm^3^; adequate hepatic and pulmonary function, no active connective tissue disorders; Karnofsky Performance Status of 70; minimum age of 18; and ability to provide a written informed consent.

### SABR technique

The SABR technique used has been reported in detail in [18, 20]. The patients were immobilized with a thermoplastic body mask, including (for liver) a Styrofoam block for abdominal compression. A contrast-free computed tomography (CT) scan were acquired for all patient and the 3 phases contrast-enhanced CT were acquired for patient with hepatic metastases. The 4-dimensional CT (4D-CT) imaging was performed in all patients with lung metastases and in 11 patients (30.5%) with hepatic metastases because a respiratory excursion was greater of 5 mm. In most of the patients, planning CT images were co-registered with magnetic resonance imaging (MRI) or positron emission tomography (PET) to better identify the gross tumor volume (GTV). The clinical target volume (CTV) was defined as equal to the GTV. In all patients who underwent 4D-CT scan, an internal target volume (ITV) was defined as the envelope of all GTVs in the different respiratory phases. The planning target volume (PTV) was generated from either the GTV or the ITV by adding an isotropic margin of 5 mm from ITV or of 7-10 mm in the cranial-caudal axis and 4-6 mm in the anterior-posterior and lateral axes from CTV.

The risk-adapted dose prescription was according to lesion site and OARs constraints respect, as showed in Table 1. For liver metastases the prescription derives from the results of the phase II trial performed at the institute [18] while for the lung metastases the risk adaptive prescription scheme is derived from institutional policies inspired to the National Comprehensive Cancer Network guidelines for lung cancer. The plan objective was to cover at least 98% of the CTV (ITV) volume with 98% of the prescribed dose (V_98%_ = 98%) and for the PTV to cover 95% of the volume with 95% of the dose (V_95%_ = 95%). Planning constraints for the organs at risk were derived from the earlier studies and included for the liver metastases: V_15Gy_ (volume receiving 15 Gy ) < (total liver volume–700 cm^3^) for healthy liver. For joint lungs excluding PTV, constraints of V_5Gy_ < 30%, V_10Gy_ < 20%, V_20Gy_ < 10 were set and a mean dose <4Gy was accepted. Treatment was delivered on a Varian TrueBeam linear accelerator using a 10 MV Flattening Filter Free beam with a maximum nominal dose rate of 2400 MU/minute with the RapidArc technique.

**Table 1:** **Summary of the risk-adapted dose prescription according to lesion site and OARs constraints respect**

	Dose	Topografical Criteria		
		Distance to chest wall	Size	Distance to main bronchus
**Lung oligometastases (** **n = ** **60)**	60 Gy/3 fr (n = 6)	>1 cm	<2 cm	>2 cm
	48 Gy/4 fr (n = 54)	>1 cm	<2 cm and <5 cm	>2 cm
**Liver oligometastases (** **n = ** **52)**	75 Gy/3 fr (n = 52)		<6 cm	

### Response assessment

Tumor response was defined using European Organization for Research and Treatment of Cancer Response Evaluation Criteria In Solid Tumors (EORTC-RECIST) 1.1 [21].

Time to local progression was calculated as the time from the first day of SABR to day of first progressive disease of the irradiated lesions. Patients were observed for local control, even if distant or new liver or lung metastases developed. PFS included any intra- or extra-hepatic and pulmonary disease progression.

After conclusion of SABR, these examinations were requested 21 days after and then every 2 months. Imaging for follow-up included CT scans every 3 months and, with the same periodicity, PET-CT was also available for a subgroup of 54% of patients. Acute and late toxicity were scored by the Common Terminology Criteria for Adverse Events 3.0. Any increase in grade from baseline was considered toxicity related to the treatment. RILD was defined by Lawrence’s criteria.

Kaplan–Meier method was used to generate the actuarial LC, OS and PFS curves. Log rank test was used for group comparison. All calculations were performed using SPSS version 13.0 (SPSS Inc., Chicago, Illinois). Univariate analysis was used to correlate morphologic and clinical factors to LC , OS and PFS and statistical significance was accepted for *p*-values of < 0.05.

## Results

### Patients and treatments characteristics

Eighty-two patients for a total of 112 single-site metastases were analyzed. Mean age was 68 years (range, 40–87years). Median follow-up was 24 months with range from 3 to 47 months. Five patients had a short follow-up (less than 6 months) because of early death. The summary of patients and treatment characteristics are reported in Table 2. Forty- two patients were treated for a total of 52 liver lesions; 41 patients were irradiated for a total of 60 lung lesions.

**Table 2:** **Patients characteristics**

Patients number	82
Mean age (range) y	68 (40–87)
Sex (M:F)	62:21
Primary	
Colon	58 (71%)
Rectum	24 (29%)
TNM Primary Classification	
T1	2 (3%)
T2	17 (21)
T3	57 (70%)
T4	6(7%)
N0	39 (48%)
N1-2	43 (52%)
M1	23 (28%)
Only liver	42 (51%)
Only lung	40 (49%)
Timing of liver metastases	
Synchronous	23 (28%)
Metachronous	59 (82%)
DFI ≤ 12 months	14 (24%)
DFI > 12 months	45 (76%)
Previous local treatments	
Surgery	33 (40%)
RFA or other	7 (9%)
Systemic treatments	
Pre-SBRT chemotherapy	78 (95%)
Post-SBRT chemotherapy	20 (24%)
Time of SBRT since diagnosis	
<12 mo	7 (8%)
>12 mo	75 (92%)
No. of prior systemic treatment regimens	
0	4 (5%)
1	15 (18%)
2	25 (30%)
3	23 (28%)
>4	15 (19%)
Presence of stable extrahepatic and pulmonary metastatic disease at diagnosis	
Yes	27 (33%)
No	55 (67%)
Number of lesions treated	112
Number of lesions for patients	Tot Liver Lung
1	61 (74%) 35 (83%) 26 (65%)
2	13 (16%) 4 (10%) 9 (23%)
3	8 (10%) 3 ( 7%) 5 (12%)
Mean volume (range) [cm^3^]	
CTV	20.3 ± 24.09 (1.0-140.3)
PTV	60.60 ± 42.16 (6.3-980.13)

In most of patients (92%) the PFS calculated from diagnosis of metastatic disease to SABR time was >12 months. Number of treated lesions was 1 in 61 (74%) patients, 2 in 13 (16%) patients and 3 in 8 (10%) patients. Mean lesion size was 3.3 cm (range 1.1 – 5.0 cm). Prescription dose ranged between 48 and 75 Gy in 3 or 4 consecutive fractions and was performed according to metastases site and organs at risk (OARs) constraints (Table 2). For 58 lesions the prescription dose was ≥ 60 Gy (6 lung metastasis and all 52 liver metastases), for the remaining 54 lung lesions the prescription dose was <60 Gy.

### Local control, progression free survival and overall survival

**Figure 1:**
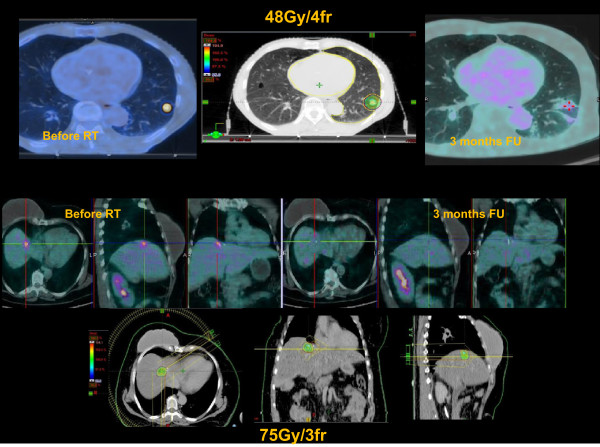
**Examples of complete response in two patients with liver and lung metastases.**

**Figure 2:**
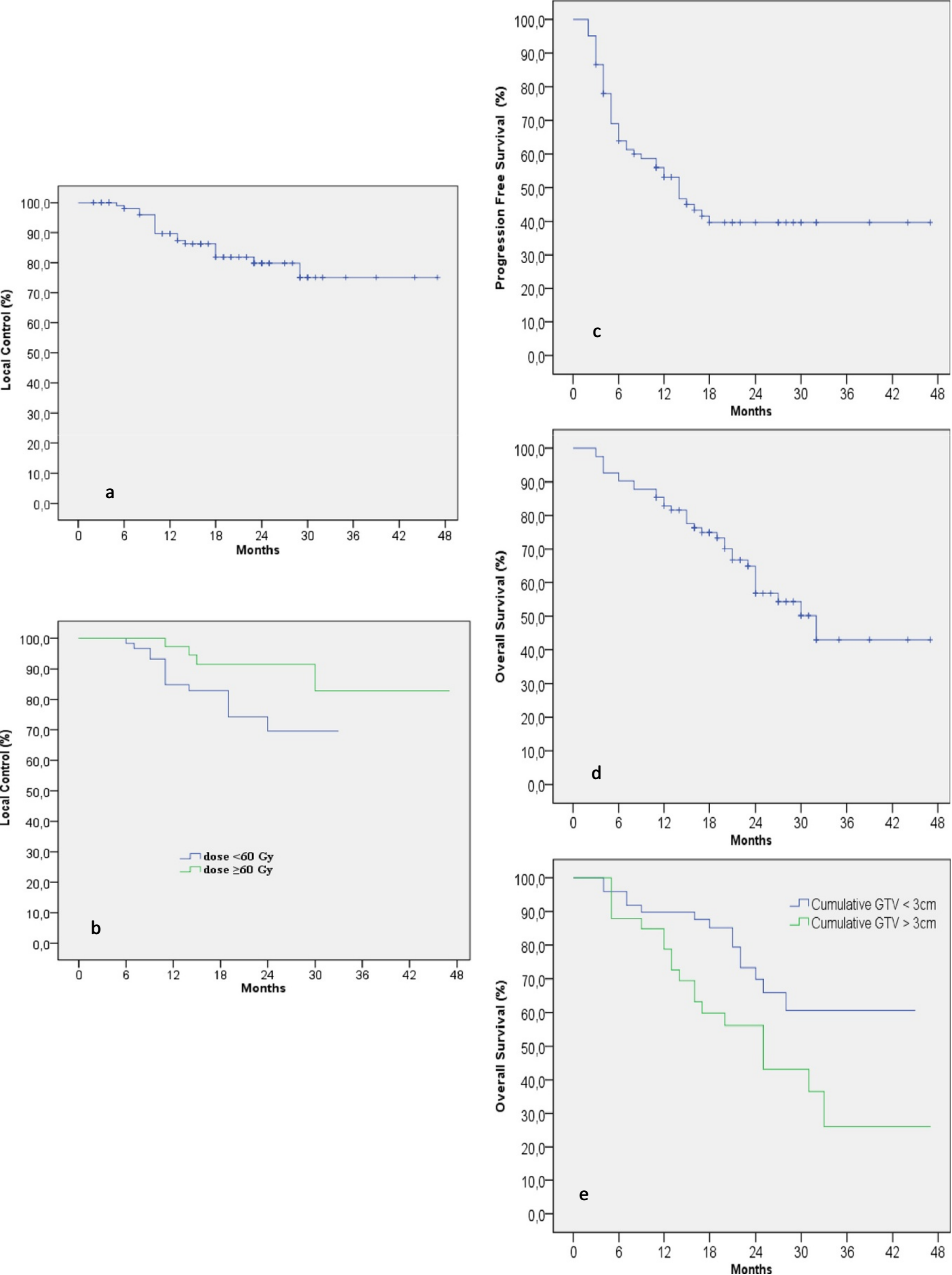
**Local control (a and b), Progression free survival (c) and Overall survival (d,e) curves.**

Figure 1 shows a complete response at 3 months FU with PET imaging in two patients with liver and lung metastases. One, two and three years LC rate was 90%, 80% and 75%, respectively (Figure 2a). Complete response was achieved in 44 (39%) lesions, partial response in 28 (25%), stable disease in 22 (20%) and progression disease in 18 (16%). The patterns of local response according to site of metastases is showed in Table 3.

**Table 3:** **Patterns of local response**

Pattern of local response	Liver metastases (n = 52)	Lung metastases (n = 60)
In-field response		
Complete response (CR)	22 (43%)	22 (37%)
Partial response (PR)	17 (32%)	11 (18%)
Stable disease (SD)	8 (15%)	14 (23%)
Progressive disease (PD)	5 (10%)	13 (22%)

Five patients for a total of 5 lesions (4%) developed infield liver recurrence at 5, 10, 13, 14 and 29 months, with a median time to liver local progression of 17 months. No correlation was observed between LC and PTV or CTV coverage (also in the cases with relatively low minimum dose to PTV). The minimum dose for the 5 recurrent patients ranged from 65.2 to 68.3 Gy (87 to 91% of the prescription). Six patients for a total of 13 lesions developed lung recurrence: 1 lesion at 6 months, 2 at 8 months, 5 at 10 months, 1 at 13 months, 3 at 18 months and 1 at 23 months, with a median time to lung local progression of 7 months. Also for these cases no correlation between LC and CTV or PTV coverage was detected.At subgroup analysis, the LC at 1,2 and 3 years, was 85%, 75% and 70% for lung metastases and 95%, 90% and 85% for liver metastases, respectively, even though difference was not statistically significant (p < 0.09). The difference in LC between the subgroup of lesions treated with dose ≥60 Gy (n = 58) and those irradiated with dose <60 Gy (n = 54) was statistically significant, with a 1,2 and 3 years LC of 97%,92% and 83%, respectively, for the higher ablative dose, compared to 85%, 70% and 70%, respectively, for the lower dose (p < 0.04), as showed in Figure 2b. No correlation with cumulative GTV dimensions, number of lesions or other factors was detected.

Forty-five (55%) patients presented with a progression disease. Patterns of progression are shown in Table 3. Median progression-free survival (PFS) was 14 months, with a PFS rate of 56% at 1 year and 40% at 3 years (Figure 2c), without correlation with the site and prescription dose of irradiated metastases (p < 0.48 and p < 0.56, respectively).

Fifty-two patients (63%) were alive at the time of analysis. Twenty-four (29%) died for cancer specific-causes, whereas 6 (7%) died of other causes. Median OS was 32 months. Actuarial OS rate at 1,2 and 3 years was 85%,65% and 43%, respectively (Figure 2d). Univariate analysis showed a correlation between OS and cumulative GTV > 3 cm (*p* < *0.024*), as showed in Figure 2e, but not with the other analyzed prognostic factors ( i.e. prescription dose, number and site of lesions, synchronous or metachronous metastases, disease free interval, in case of metachronous disease, greater or lesser than 12 months, presence of extra-hepatic and extra-pulmonary metastatic disease at the time of diagnosis, previous chemotherapy regimens), as shown in Table 4. Disease specific survival did not significantly differ from OS because most of the patients died for cancer related causes (only 4 patients died for other causes).

**Table 4:** **Prognostic factors affecting LC and OS rates on univariate analysis**

Factors	Lesion (n°)	LC (rates)			***p***value	Patients (n°)	OS (rates)			***p***value
		1 years	2 years	3 years			1 years	2 years	3 years	
**Site of irradiated metastases**										
**Lung**	60	85%	75%	70%	*0.095*	40	87%	68%	58%	*0.34*
**Liver**	52	95%	90%	81%		42	78%	61%	44%	
**Cumulative GTV**										
**< 3 cm**	-	-	-	-	-	47	90%	70%	61%	*0.02*
**> 3 cm**						35	73%	56%	26%	
**SBRT dose**										
**≥ 60 Gy**	58	97%	92%	83%	*0.043*	46	80%	57%	38%	*0.69*
**< 60 Gy**	52	85%	70%	70%		36	86%	54%	-	

### Toxicity

Fifty-four patients (70%) developed G2 acute toxicity. The most frequent side effects were fatigue (60%) and transient hepatic transaminase increase (25%), for liver metastases treatment. No toxicity of grade 3 or greater was observed. No patients developed RILD, chest pain or rib fracture.

## Discussion

Although the role of oligometastases ablation was often controversial, evidence was provided to support the efficacy of metastatic resection [5–11, 22]. Liver and lung are a common sites of progression in CRC, with an incidence of 30-70% [5]. The modern chemotherapy regimens have improved the prognosis of this oligometastastic patients, but the surgery has allowed the major results in terms of long-term outcomes [23]. Surgical resection improves OS, with 1- and 5-year rate of 90-95% and 30-60%, respectively for liver metastases and with 1- and 5-year OS rate of 85-90% and 38-50% respectively, for lung metastases [6–11, 22]. An increased long-term cancer-specific survival at 10 years after resection was recently demonstrated [22]. About 80-90% of metastatic patients, however, are not suitable for resection because of technical difficulties, unfavorable tumor factors or patients co-morbidities [23, 24]. RFA is a valid alternative to surgery, with a LC rate of 90-98% at 1 year, OS rates at 1–2 and 5-year of 87%-70% and 34%, respectively, and median OS of 25 months [12–14]. Efficacy of local therapies is acceptable in presence of small lesions with diameter <3 cm and distant from vascular or biliary structures. An effective and safe alternative therapeutic option is necessary in about 60-80% of CRC oligometastatic patients, which can benefit from locally ablative therapy, as they are probably never fit to surgery.

SABR represents such an alternative for tumor ablation. Different from conventional radiotherapy, SABR entails precise delivery of high-dose in few fractions, with a complete tumor ablation and maximal normal-tissue sparing. Prospective studies have supported the use of SABR in oligometastatic patients [15]. The rationale of oligometastatic ablation with SABR consists of a very complex net of factors [25], to which the impact of immune-modulation is added [26].

Many authors have shown the efficacy of SABR as a local treatment of oligometastases in liver, lung and lymph nodes from different primary cancers [16–19]. However, only few study are focused on the SABR for inoperable oligometastases from CRC, with a limited number of patients (ranged between 20–59) and lesions (ranged between 31–78) treated, as shown in Table 5 [27–30].

**Table 5:** **Published study on SBRT for oligometastases from CRC**

Author, design study, (reference)	Patients (n)	Lesions (n)	Dose (Gy/ fr)	FUP (m)	LC (%)		OS (%)		Median PFS (m)	Acute Toxicity ≥G3
					1-year	2-yeras	1-year	2-years		
**Hoyer, Phase II (27)**	44	-	45 Gy/3 fr	4.3 y	90%	79%	67%	38%	6.5	48%
**Van der Pool, Phase I-II (28)**	20	31	37.5 Gy/3 fr	26 m	100%	74%	100%	83%	11	10%
			45 Gy/3 fr							
			(2 pts)							
**Kang, Retrospective (29)**	59	78	36–51 Gy/3 fr	32 m	85%	66%	82%	66%	-	3%
			14 Gy/1 fr							
**Bae Retrospective (30)**	41	50	45–60 gy/3 fr	28 m	100%	85%	85%	70%	-	7%
**Current Study**	82	112	48 Gy/4 fr	24m	85%	70%	85%	65%	14m	0%
			60–75 Gy/3 fr		97%	92%				

In the present prospective analysis, 82 consecutive patients with a total of 112 single-site oligometastases from CRC were treated with an ablative radiation dose ranged between 45–75 Gy in 3 or 4 fractions.

All patients presented a single site of metastatic disease, liver or lung , and a maximum of three lesions treated. Median follow up was 24 months, 1,2 and 3-year LC rates were 90%, 80% and 75%, respectively. Univariate analysis showed a statistically significant improvement of LC in the subgroup of lesions treated with a prescription dose ≥ 60Gy. This is in agreement with several studies focused on SBRT for liver metastases. Lee et al. [17] confirmed the correlation between local control and higher prescription dose, specially for lesion larger than 3 cm, as showed by Rusthoven et al. [16]. A pooled analysis on SBRT for CRC liver metastases by Chang et al. [31] confirmed the better local control for lesion treated with higher prescription dose and suggested the use of a total dose > 48 Gy for a 3 fractions regimen of SBRT.

Improvement in LC is more evident after 1 year of FU and confirms the importance of the use of ablative doses in this subset of long- survival CRC oligometastatic patients.

In our study, LC is not correlated to the cumulative GTV (larger or smaller than 3 cm in diameter) when a higher prescription dose is administered, according to our results on SBRT for liver metastases [18]. This suggests the utility of escalations dose of radiation in the absence of severe complications. The improvement in LC is more evident, in this study, after 1 year of FU and confirms the importance of the use of ablative doses in this subset of long-surviving CRC oligometastatic patients.

Median OS was 32 months. Although the FU is still short for a data comparison with the surgery and RFA, these results are considered promising. This remark is strengthened by univariate analysis, which showed a correlation between OS and cumulative GTV > 3 cm (p < 0.02) and a median OS of 44 months for a subgroup of patients with lower cumulative GTV. OS was not influenced by other prognostic factors (synchronous or metachronous presentation, DFI, extra-hepatic or extra-pulmonary disease, previous chemotherapy regimens), according to data published on SBRT. These data seems to be related to the careful selection of these oligometastatic patients, most of which (90%) presented a time-interval from diagnosis to SABR > 12 months and a stable oligometastatic disease. Correlation between OS and cumulative GTV, suggested that it is important to perform SBRT in oligometastatic patients before a wider spreading of disease.

Although median follow up of this study was 24 months, results seem to encourage the use of SABR in the treatment of CRC oligometastatic patients not eligible for surgery and/or RFA because of tumor size and/or location and patient comorbidities. This study has shown that SABR, with a low toxicity profile, is a safe and effective therapeutic option also for “frail” and elderly patients.

## Conclusions

SABR is a safe, non-invasive and effective therapeutic option for unresectable colorectal oligometastases and allows to achieve promising rates of LC and OS. Dose higher 60 Gy are recommended to improve LC.
